# Liquid crystal-assisted optical biosensor for early-stage diagnosis of mammary glands using HER-2

**DOI:** 10.1038/s41598-023-31668-8

**Published:** 2023-04-26

**Authors:** Mehri H. Pourasl, Ali Vahedi, Habib Tajalli, Balal Khalilzadeh, Farzaneh Bayat

**Affiliations:** 1 Department of Physics, Tabriz Branch, Islamic Azad University, Tabriz, Iran; 2 Biophotonic Research Center, Tabriz Branch, Islamic Azad University, Tabriz, Iran; 3 Stem Cell Research Center (SCRC), Tabriz University of Medical Sciences, Tabriz, 51664-14766 Iran; 4 Department of Physics, Azarbaijan Shahid Madani University, Tabriz, Iran

**Keywords:** Breast cancer, Optical physics, Analytical chemistry

## Abstract

Breast cancer (BC) is one of the most commonly diagnosed cancers and the second leading cause of cancer mortality among women around the world. The purpose of this study is to present a non-labeled liquid crystal (LC) biosensor, based on the inherent feature of nematic LCs, for the evaluation of BC using the human epidermal growth factor receptor-2 (HER-2) biomarker. The mechanism of this sensing is supported by surface modification with dimethyloctadecyl [3-(trimethoxysilyl) propyl] ammonium chloride (DMOAP) encouraging the long alkyl chains that induce a homeotropic orientation of the LC molecules at the interface. To enhance the binding efficacy of more HER-2 antibody (Ab) on LC aligning agents, a simple ultraviolet radiation-assisted method was also used to increase functional groups on the DMOAP coated slides, thereby improving binding affinity and efficiency onto HER-2 Abs. The designed biosensor makes use of the specific binding of HER-2 protein to HER-2 Ab and disruption of the orientation of LCs. This orientation change leads to a transition of the optical appearance from dark to birefringent, enabling the detection of HER-2. This novel biosensor exhibits a linear optical response to HER-2 concentration in the wide dynamic range of 10^–6^–10^2^ ng/mL, with an ultra-low detection limit of 1 fg/mL. As a proof of concept, the designed LC biosensor was successfully investigated for the quantification of HER-2 protein in patients suffering from BC. Owing to the sensitivity, selectivity, and label-free detection, this biosensor may amplify the application of LC-based biosensors for the detection of most types of cancers.

## Introduction

Breast carcinoma is the most frequent type of cancer in females with an estimated nearly 900 thousand deaths per year and also it is the second most prevalent cancer in the world^1–7^. The human epidermal growth factor receptor 2 (HER-2) is the best-known predictive and prognostic marker in breast cancer (BC). This protein is amplified in about 15–20% of BCs and transmits signals mediating aggressive tumor behavior, while also it provides a tremendous opportunity to develop targeted treatments^8–10^.

Despite decades of basic and clinical investigation and promising new treatment options, BC morbidity continues to rise among women, infecting one in 20, globally^11^. Accordingly, efforts aimed at early diagnosis of this kind of cancer can potentially improve the chance of survival and patient therapy outcomes^12–16^. The fluorescence in situ hybridization (FISH), polymerase chain reaction (PCR)^17^, and immunohistochemical methods are conventional diagnosis assays for HER-2 determination. Due to some restrictions such as the lack of sensitivity, time-consumption, complexity, and expensiveness in mentioned methods, alternative technologies have been widely studied to more sensitively and effectively detect the HER-2 biomarker^18,19^. These include electrochemical^20–25^, optical^26–31^ and piezoelectric biosensors^32,33^. However, these techniques also have their own cons and pros.

Taking advantage of the properties such as the high sensitivity, simplicity of use, robustness, detection of multi-analyte, automatized microfluidic devices and ability to be incorporated on a single chip, optical platforms have lent themselves as analytical methods with wonderful versatility for variant biosensing applications^28,34–36^. Hence, optical biosensors are good alternatives as a non-invasive technique to presently used cancer diagnosing methods^19,37,38^. Among optical sensing approaches, liquid crystal (LC) biosensors have become an emerging research field for cancer biomarkers evaluation in recent years^39–43^. LCs are a prospective branch of excellent sensing and phase transition materials that exist between isotropic liquids and highly ordered solid crystals, hence LCs are mentioned as intermediate phases or mesophases^44–46^. They have the ability to flow like fluids and also exhibit anisotropic peculiarities such as the birefringence of crystalline materials^47,48^.

Optical, anchoring and elastic properties of LC materials play a fundamental role in LC-based detection. Anisotropic optical properties of LC molecules result in birefringence^49^. This property creates different refractive indices that light encounters due to its polarization direction. This phenomenon causes alteration in the phases and the states of polarization detected by the signal transducer. LC platforms are capable of tuning the polarization of light caused by birefringence and transmuting this into an optical texture, which can be monitored by polarized-light optical microscopy (POM). On account of the interference of two orthogonal rays, the visible optical images of LCs can be perceived through crossed polarizers. Moreover, when the director orients parallel to the light propagation direction, the optical signal will be lost. In other words, the linearly-polarized beam cannot be observed in the case of the homeotropic orientation of LCs, whereas does not permit birefringence and creates a dark optical appearance^50,51^.

LCs have very weak anchoring energy on surfaces (in nematic LCs from 10^–3^ to 10^–7^ mJ/m^2^ ^48^) which makes them ultra-sensitive to external variables^52^ at the interface and topography changes. These responsive materials can disturb the preferred orientation of LC molecules. Furthermore, the high mobility and elastic nature of the LCs director field can further trigger the amplification of the surface-induced ordering transition to a distance of approximately 100 μm into the bulk LCs^48,50^. At the same time, LC materials can prepare a signal directly upon molecular binding without the need for any secondary probes carrying a label that generates the signal^53^.

According to the sensing interfaces, the three categories of LC-based biosensing platforms have been designed including LC–solid interface^54^, LC–aqueous interface^55^, and LC–droplet interface^56^. The proffered biosensor in this paper is based on the LC–solid interface format, which is simple in design and also able to realize array structures. Overall, the mechanism in these biosensors essentially affords the LC molecules' reorientation due to the influence of targets. This permits LCs to react to biological or chemical matters, and the response signals can be transformed into measurable optical/electrical indicators. Over the past few years, several researchers have explored the application of LCs as optical probes in biosensing systems because of the unique properties possessed by LCs and achieved fast growth^57–64^.

In the present work, a label-free LC-based biosensor for the determination of HER-2 cancer biomarkers was designed and fabricated. The recognition principle of this biosensor is based on a correlation between HER-2 protein detection sensibility and the optical peculiarities of LCs. Accordingly, HER-2 protein is reacted with its antibodies (Abs) immobilized on glass slides covered with Dimethyloctadecyl [3-(trimethoxysilyl) propyl] ammonium chloride (DMOAP) as the aligning agent. This binding can dramatically disturb the orientation of LC molecules and creates birefringence with bright optical images which can be observed through POM. Herein, the binding efficiency between the HER-2 Abs and DMOAP was improved by ultraviolet (UV)-assisted radiation. Following the irradiation, the density of hydrophilic functional groups was amplified^60^. In addition, the covalent bonding between the HER-2 Ab and UV-modified DMOAP was enhanced via 1-ethyl-3-(3 dimethylaminopropyl) carbodiimide hydrochloride and N-hydroxysuccinimide (EDC/NHS) coupling agents. The EDC/NHS based crosslinking of Abs significantly ensures robust immobilization and improved the stability of the designed biosensor^65,66^. By incorporating both procedures, it is anticipated to meliorate the accuracy, repeatability, and sensitivity of LC biosensors, as well as their potential in real clinical samples.

## Materials and methods

### Materials

Glass microscope slides (76 mm × 26 mm) were obtained from Superior Company (Germany). DMOAP (42 wt.%) was bought from Sigma-Aldrich. 4-Cyano-4′-pentylbiphenyl (5CB) as nematic LC was purchased from the Institute of Chemistry of the Military Technical Academy, Warsaw, Poland. HER-2 Ab (ab214275) and HER-2 protein (ab60866) were obtained from the Abcam company. The solution of HER-2 protein was prepared using the dissolving 250 mg/mL of the protein in deionized (DI) water. Working standard solutions applied for the calibration curve was prepared by diluting the stock protein solution with DI. The 200 μg/mL of HER-2 Ab was applied in all assays. EDC/NHS were acquired from Merck. Phosphate-buffered solutions (PBS) were provided in room temperature (RT) and including NaCl (8.000 g/L), KCl (0.201 g/L), KH_2_PO_4_ (0.231 g/L), Na_2_HPO_4_ (3.581 g/L), pH = 7.4.

### The fabrication process of LC cells

#### Pretreatment of glass slides

First, the glass slides were cut into 1 cm × 7.5 cm pieces and then incubated in the freshly prepared piranha solution (containing 70% sulfuric acid, and 30% hydrogen peroxide) for about 1 h (h) at 80 °C to remove all organic matters and increase the hydrophilicity of the surface. The piranha-treated glass substrates were rinsed thoroughly with DI water. Then, they were heated in an oven at 110 °C for at least 3 h after dehumidifying by a stream of nitrogen gas. The glass slide surfaces were functionalized by hydroxyl groups (–OH) after treatment.

#### Decoration with DMOAP

The piranha-treated glass slides as the both covering (the upper slides) and bottom layer were submerged in a 1% (v/v) DMOAP aqueous solution for 30 min at RT. Then, the slides were rinsed several times with DI water to eliminate the unbound DMOAP from the surface. Subsequently, the DMOAP decorated glass slides were dried with nitrogen flow and baked in a 100 °C oven for 3 h to permit the cross-linking of DMOAP. The alkoxy groups (C_2_H_5_O–) in DMOAP react with –OH groups of piranha treatment. Thus, the stable grafting of DMOAP is formed onto the surface. To provide the UV-activated DMOAP monolayer, the DMOAP coated glass substrates were exposed to UV irradiation at 405 nm with an intensity of 10 mW∕cm^2^ for 15 min.

#### HER-2 Ab immobilization

First, a desirable concentration of HER-2 Ab was provided with DI water. Afterward, 50 µL of the 1:1 EDC/NHS ratio was gotten by the formerly prepared stock solutions and mixed with 25 µL of Ab solution at RT. Thus, EDC/NHS activated HER-2 Ab was bound to the UV-modified DMOAP glass slides at 4 °C overnight. The EDC/NHS coupling system was employed by crosslinking HER-2 Ab on the functionalized surface. Then, the substrates were rinsed successively with PBS solution (pH = 7.4) and DI water by tilting the glass slides, to remove the unbinding HER-2 Abs, and finally, dried at RT.

#### Specific binding of HER-2 protein and HER-2 Ab

A volume of 50 µL of different concentrations of HER-2 protein solutions was poured on glass slides which were immobilized with HER-2 Ab and stored at 4 °C for 3 h. Then, the substrates were washed sequentially with PBS solution (pH = 7.4) and DI water to remove unbound agents, and eventually dried at RT.

#### Assembling the LC-based biosensing platform

The LC biosensors were constructed by pairing the UV-modified DMOAP coated glass slides bearing the HER-2 Ab array and HER-2 protein as the substrate glass slides and upper glass slides which were decorated with DMOAP. The two slides were face to face separated by two strips of spacers (thickness = 7 µm). Thus, a gap between two glass slides was created and then they were sealed with epoxy glue.

5CB was heated at $\sim \hspace{0.17em}$40 °C for 10 min (isotropic phase of 5CB), afterward, a small amount of 5CB was introduced into the gap between two glass slides utilizing capillary force. Subsequently, the optical cells were steadily cooled at approximately 25 °C (5CB, nematic phase from 22.00 to 35.5 °C) before characterization. The cell-based biosensors were observed with a POM (XPT-7 polarizing microscope) equipped with crossed polarizers in the transmission mode. The optical images were taken by an EOS 250D digital camera mounted on the POM.

### Fourier-transform infrared (FTIR) spectroscopy

FTIR spectra of unmodified DMOAP and UV-modified DMOAP were taken by a Bruker Tensor 27 FTIR spectrometer in the transmission mode. For this purpose, methanol solution containing 1% (v/v) DMOAP was decorated on the cleaned glass slides, followed by heating at 100 °C for 3 h. Then the glass slides were irradiated under UV light at 405 nm with the power of 10 mW∕cm^2^ for 0–20 min. The slides were subsequently submerged in methanol, and the FTIR spectrum of this solution was acquired in the range of 1000–4000 cm^−1^.

### Ethics approval and consent to participate

All patients were asked to complete the informed consent. All procedures of this study were approved by the Local Ethics Committee of Tabriz University of Medical Sciences (IR.TBZMED.VCR.REC.1400.150). All procedures were done under the declaration of Helsinki.

## Results and discussion

### Sensing mechanism and detection principle of the designed LC biosensor

**Figure 1:**
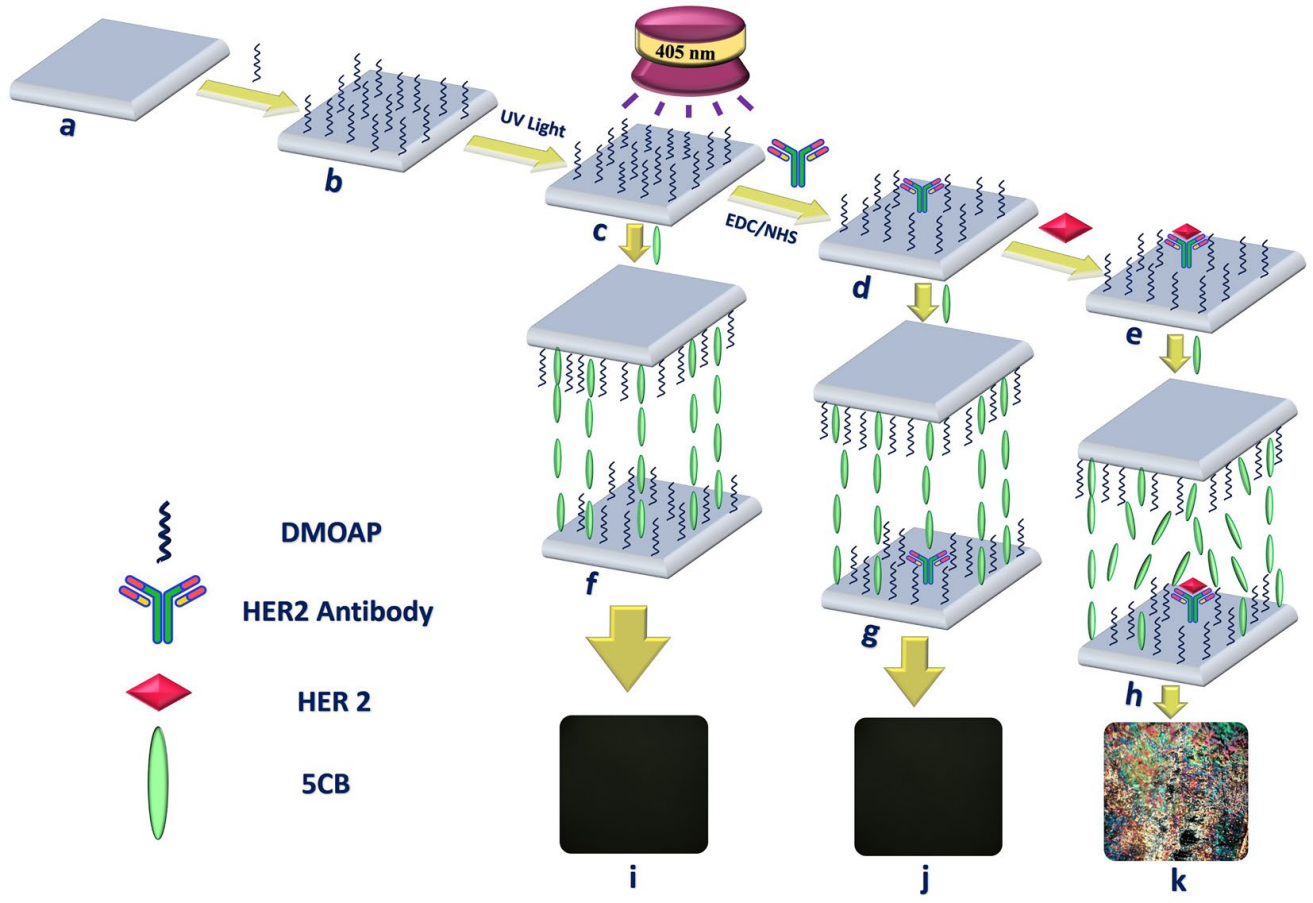
Schematic preparation steps of LC-based biosensor for HER-2 detection; (**a**) the piranha-treated surface; (**b**) DMOAP-functionalized surface; (**c**) UV-activated DMOAP coated surface; (**d**) immobilization of HER-2 Ab; (**e**) HER-2 specific binding to the HER-2 Ab; (**f**) homeotropic alignment of LC 5CB molecules in the optical cells without biomolecules; (**g**) perpendicular orientation of LC 5CB in the optical cells with HER-2 Ab; (**h**) orientation disruption of 5CB in the optical cells with HER-2 protein; (**i**) POM appearance of LC cells in the absence of HER-2 Ab; (**j**) POM appearance of LC cells in the absence of HER-2 protein; (**k**) POM appearance of LC cells in the presence of HER-2 protein (Designed by PowerPoint 2016 and SolidWorks 2018).

The sensing mechanism of the proposed LC biosensor for HER-2 biomarker detection is provided schematically in Fig. 1. The glass slides pre-treated with piranha liquid (Fig. 1a) were decorated with DMOAP and used as covering and lower glass slides (Fig. 1b). Then, for further grafting of HER-2 Ab, the DMOAP modified surface was exposed to UV irradiation which will generate hydrophilic functional groups on DMOAP (Fig. 1c). LC molecules form a vertical alignment fixed by the interaction with DMOAP as the orientation agents with long alkane thiol. In the absence of biomolecules and also with the synergistic effect of the DMOAP on the covering surface (Fig. 1f), the LC cell displays a uniformly dark optical output (Fig. 1i). It should be noted that the stabilization and enhancement of HER-2 Ab binding were contributed not only to the promoted hydrogen bonds between the UV-modified DMOAP oxygen functional groups and the polar functional groups such as –COOH and amid (–NH_2_) of biomolecules, but also possibly to the covalent bonds using the EDC/NHS as a crosslinking regent. The immobilized HER-2 Abs (Fig. 1d) are deficient to affect the homeotropic orientation of the LC molecules (Fig. 1g), suggesting that the HER-2 Ab couldn't change the modified surface topography in contact with the LC molecules and the POM images remained dark, too (Fig. 1j). However, in the presence of HER-2, target particularly interacts with HER-2 Ab immobilized on the substrate to form an “Ab-antigen” complex (Fig. 1e). This complex lead to the distorted alignment of LCs as the result of their larger sizes (Fig. 1h). The visible color optical image of LC, therefore, could be observed by using the POM (Fig. 1k).

Different concentrations of target protein on the modified surface induced variant degrees of LC molecules disruption that cause a differential brightness in POM images. Furthermore, quantitative analysis of HER-2 concentration can be done.

### Effect of UV irradiation on the DMOAP-coated slides

The covalent binding of capture Abs provides sturdy and stable immobilization and improves density and Abs orientation results on the glass surface. However, to meet further immobilization affinity of antibodies toward DMOAP, the modification of DMOAP monolayer with UV light has been investigated, too.

**Figure 2:**
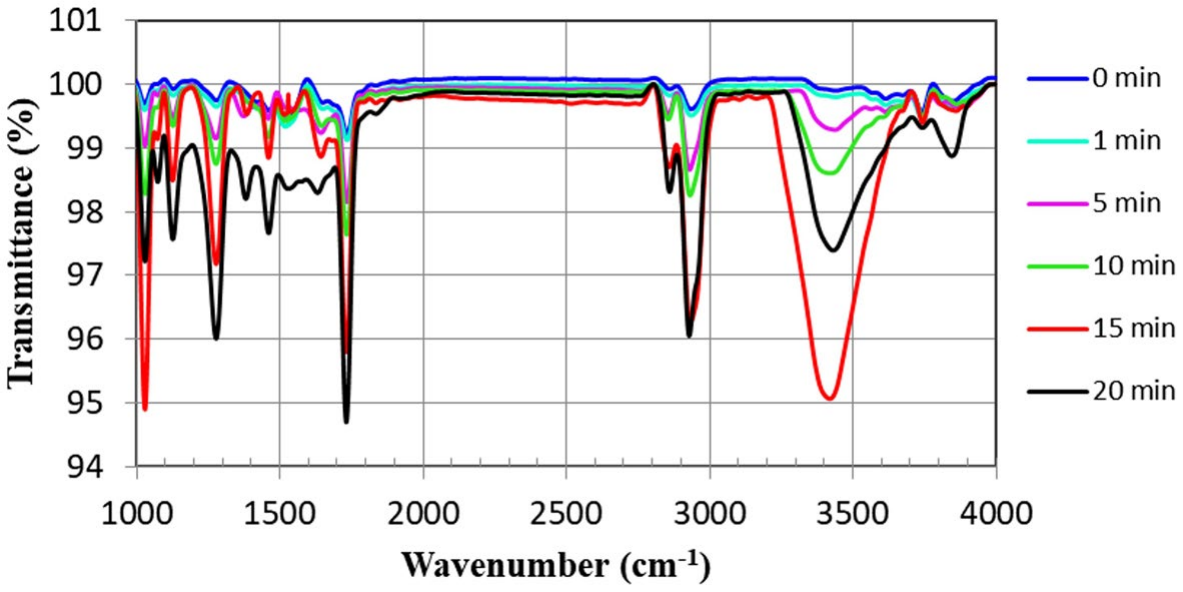
FTIR analysis of unmodified DMOAP and UV-modified DMOAP samples. The FTIR spectra were recorded in the transmission mode with an FTIR spectrometer.

Since the unmodified DMOAP- coated surface does not have any reactive groups and just has a hydrophobic alkyl group at its terminus, activating of DMOAP by UV light provides functional groups such as –COOH, aldehyde (–CHO), –OH, and carbonyl (C=O)^67,68^. Under various UV exposure times, the kinds of functional groups generated on UV-modified DMOAP samples were individualized by FTIR spectroscopic analysis^69^. Figure 2 displays the FTIR spectra of DMOAP radiated glass slides. The irradiation process was done for 0, 1, 5, 10, 15, and 20 min. The band corresponding to the C–H stretching of alkane (2850–3000 cm^−1^) illustrates the alkyl terminal group of the DMOAP monolayer.

The –OH groups show their characteristic band between the 3200 and 3500 cm^−1^ regions and the other characteristic peak intensity in the 1050–1200 cm^−1^ is associated with the C–O stretching of aldehydes or alcohols, which increased by enhancing the time of UV radiation. In addition, C=O groups peak intensity in the 1600–1700 cm^−1^ region, significantly increased when UV exposure time on DMOAP is 20 min. By prolonging UV exposure time (20 min and above), some of the characteristic bands corresponding to functional groups weaken and may cause adverse effects on the immobilization of antibodies. This suggested that polar and oxidized functional groups having oxygen, that is, the origin of the hydrophilicity in DMOAP, are generated under UV-assisted surface modification of the DMOAP monolayer.

These results show that the effective UV radiation exposure time of DMOAP in all subsequent assays was confined to 15 min to provide a sufficient number of functional groups on DMOAP and may remarkably –OH groups in place of –CHO or –COOH groups.

### Optimization of the HER-2 Ab concentration

**Figure 3:**
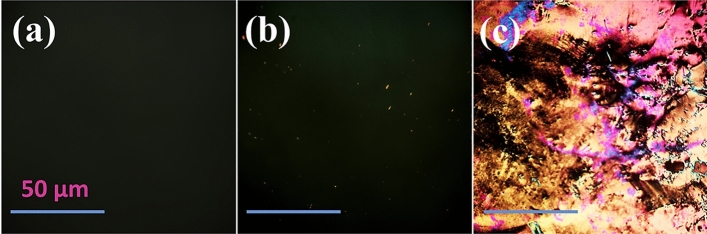
POM appearances of LC biosensor cells in the presence of variable concentrations of HER-2 Ab: (**a**) 2 µg/mL, (**b**) 200 µg/mL, and (**c**) 2000 µg/mL. Scale bar: 50 µm.

As was mentioned before, the surface topographic change is extremely related to the orientation of LC molecules and HER-2 Ab becomes a probe to bind with HER-2 that directly disrupts the orientation-ordered LCs. Thus, it is important to survey the optimal concentration of Ab that retains the homeotropic arrangement of the LC molecules. Figure 3 demonstrates the POM appearances of LC cells made from the UV-activated DMOAP-coated surfaces incubated in different concentrations of HER-2 Ab. The bright spots in the optical images disappeared when the concentrations of HER-2 Ab were equal to or less than 200 µg/mL. As shown in Fig. 3c, when the concentration of HER-2 Ab was higher than 200 µg/mL, a color image appeared. This phenomenon indicates that the LC molecules are oriented randomly. According to these results, the HER-2 Ab concentration of 200 µg/mL was chosen as the optimum concentration of Ab for all experiments to keep the signal-to-background constant. Also, the selected concentration ensures an appropriate quantity of HER-2 Ab to maximize its concentration for enhancing the performance of the biosensor.

### Detection of HER-2-based designed biosensing platform

**Figure 4:**
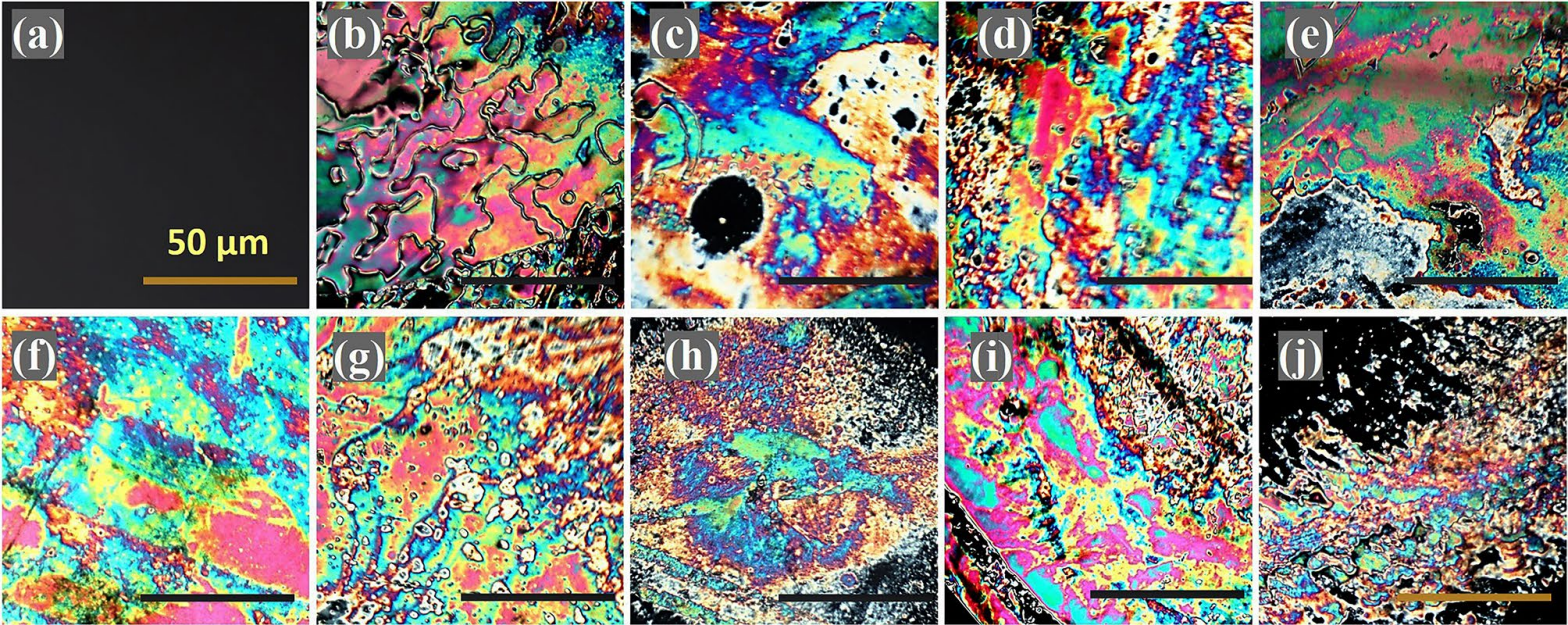
POM appearances of LC biosensors cells in response to HER-2 protein at the concentration of: (**a**) 0, (**b**) 10^–6^, (**c**) 10^–5^, (**d**) 10^–4^, (**e**) 10^–3^, (**f**) 10^–2^, (**g**) 10^–1^, (**h**) 1, (**i**) 10 and (**j**) 10^2^ ng/mL. Scale bar: 50 µm.

After acquiring the optimal Ab concentrations, to emphasize the possibility of applying the presented biosensor for the target HER-2 protein detection, the various concentration of the HER-2 protein was incubated with LC-prepared cells. As presented in Fig. 4, by increasing the concentration of HER-2 from 10^–6^ to 10^2^ ng/mL, the optical images of the LC cells show more obvious interference colors. This is evidence of the HER-2 protein binding to HER-2 Ab on the surface which is capable of the homeotropic to random/tilted transition of LC molecules. As the concentration of HER-2 protein decreased from 10^–6^ ng/mL, an optical image with no obvious interference was captured, demonstrating that less than 10^–6^ ng/ml concentration of HER-2 cannot disrupt the ordered arrangement of LC molecules. From reproducibility study, all concentrations were tested three times at the identical sensing condition. The similar optical images were observed and concequently the recorded results were partly reproducible.

**Figure 5:**
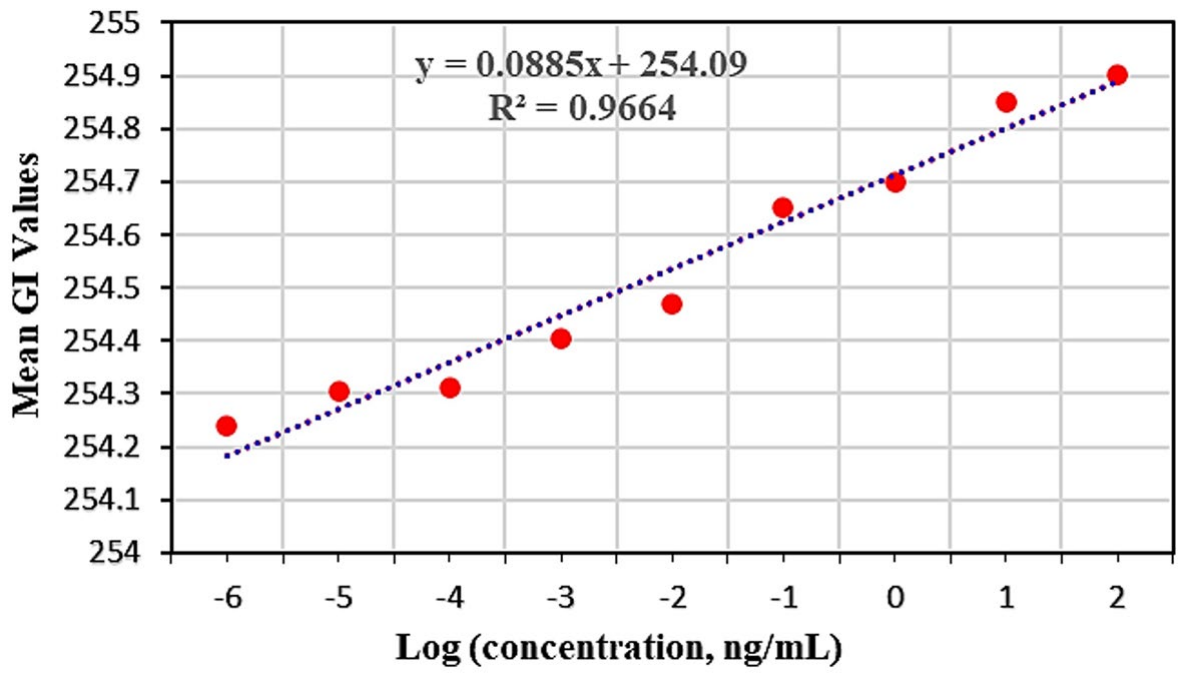
The calibration curve of the mean GI of the POM appearance via the logarithm concentration of the HER-2 protein.

To investigate the quantitative analysis of LC biosensor performance, according to the frequent measurements (3 times), the average gray-scale intensity (GI) present in the POM image was calculated using freely available ImageJ software^70^. As depicted in Fig. 5, the measured average GI calibration curve as a function of the logarithm concentration of HER-2 in a wide range from 10^–6^ to 10^2^ ng/mL showed a linear relationship with a desirable correlation coefficient (R^2^) over 0.96. Using these LC-based biosensors, the detection limit of HER-2 is obtained as about 1 fg/mL. As shown in Table 1, in contrast to other reported optical biosensors for HER-2 detection, our procedure offers relatively higher sensitivity with excellent efficiency.

**Table 1:** The comparative performance of the presented biosensor with the other optical sensing mechanism for HER-2 detection.

S. no	Biosensor structure	Type	Detection limit	Linear range	References
1	Opto-fluidic ring resonator (OFRR)	Resonator type	10 ng/mL	13–100 ng/mL	^71^
2	Optical fiber-based SPR^b^ (OF-SPR^b^)	Resonator type	9.3 ng/mL	n.r.^a^	^72^
3	Nano hole arrays/Au	SPR^b^	3.0 ng/mL	n.r.^a^	^27^
4	Taper interferometer embedded in a fiber brag grating (FBG)	Fiber type	2 ng/mL	n.r.^a^	^73^
5	Quantum dots and Au as a donor–acceptor Pair	FRET^c^	1 ng/mL	2–100 ng/mL	^74^
6	Bloch surface wave (BSW) biochip based on photonic crystal	Fluorescence	0.3 ng/mL	n.r.^a^	^75^
7	Dual signal-labeled fluorescent probe solution	Fluorescence	0.042 ng/mL	3.5–5 ng/mL	^29^
8	Ruthenium complexes and tri-propyl amine	Electrochemilu-minescence	1fM	1fM-1 nM	^28^
9	LC-solid interface on the glass substrate	LC biosensor	1 fg/mL	10^–6^–10^–2^ ng/mL	Reported here

^a^Not reported conversion.

^b^Surface plasmon resonance.

^c^Fluorescence resonance energy transfer.

### Selectivity

**Figure 6:**
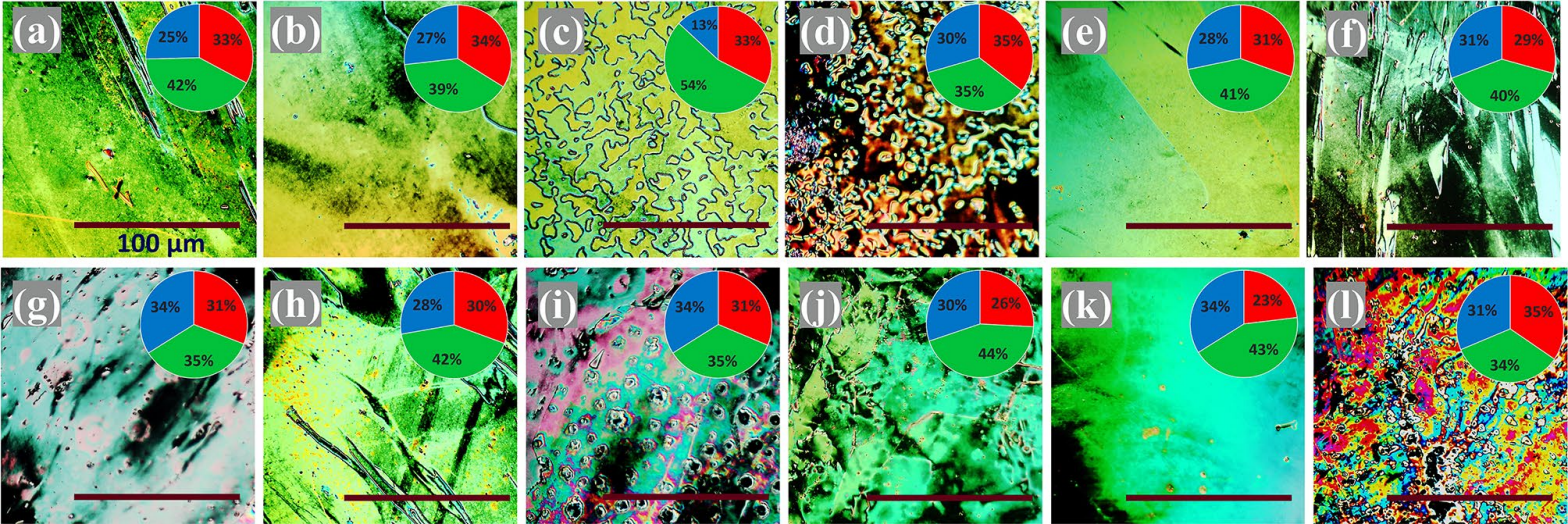
POM appearances of LC biosensor cells after incubation of HER-2 Ab immobilized surface with 0.01 IU (**a**) CA125, (**b**) CA19-9, (**c**) CEA, (**d**) CA15-3, (**e**) PSA, and (**f**) 0.01 ng/mL HER-2 + 0.01 IU CA125, (**g**) 0.01 ng/mL HER-2 + 0.01 IU CA19-9, (**h**) 0.01 ng/mL HER-2 + 0.01 IU CEA, (**i**) 0.01 ng/mL HER-2 + 0.01 IU CA15-3, (**j**) 0.01 ng/mL HER-2 + 0.01 IU PSA, at equal ratio, (**k**) mixture of proteins (0.01 IU CA125, CA19-9, CEA, CA15-3, PSA and 0.01 ng/mL HER-2 at equal ratio), and (**l**) 0.01 ng/mL HER-2. Scale bar: 100 µm.

**Figure 7:**
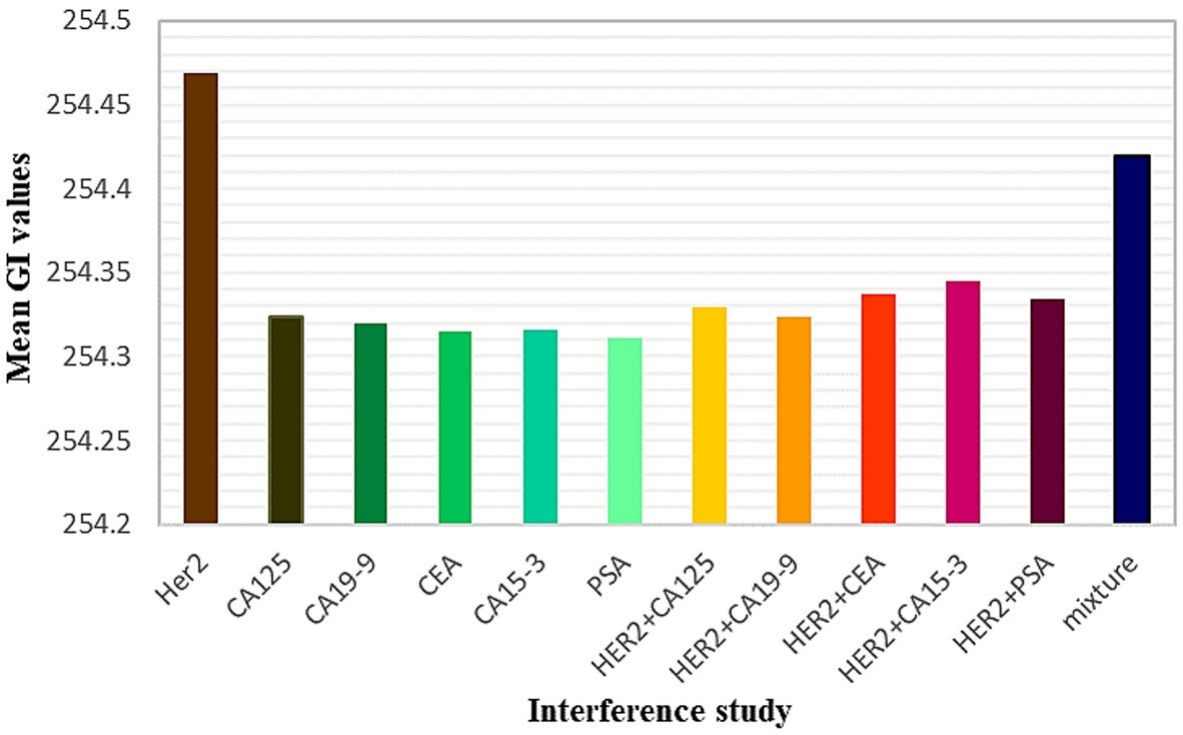
Correlated histograms of average GI of 0.01 ng/mL of HER-2 protein, 0.01 IU of other interferences proteins, the combination of HER-2 with interferences proteins, and the mixture of all proteins.

For verifying the specificity of this method, the optical signals readout of five possible interferences [prostate specific antigen (PSA), cancer antigen 19–9 (CA19-9), cancer antigen 15–3 (CA15-3), cancer antigen 125(CA125), carcino-embryonic antigen (CEA)] was evaluated under a similar experimental state. As shown in Fig. 4, the POM image of 0.01 ng/mL HER-2 displays variant interference colors, while the optical images of the above proteins, the combination of each with HER-2 and also a mixture of them (all in 0.01 IU) within 0.01 ng/mL HER-2 at an equal ratio, have almost monochrome optical textures (Fig. 6). To have a more quantitive point of view the RGB (red, green and blue) percentage values are presented in pie charts for each image in Fig. 7. It can be concluded that for the more equal percentage of RGB values, the more successful are the proteins in disrupting the liquid crystal molecules. Thus, much more color varieties can be observed in optical images. Figure 7 confirms that the highest amount of disruption is for the case of applying only HER-2 proteins. Furthermore, the ratios of optical signal responses (Mean GIs) are significantly lower compared to that of HER-2 as represented in the average GI (Fig. 7). These results revealed that LC-based biosensors present enough selectivity toward HER-2.

### Real sample analysis and stability investigation

**Figure 8:**
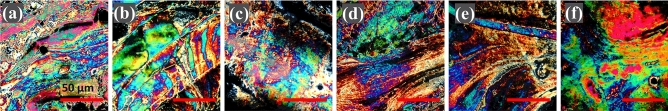
POM appearances of LC biosensor cells after incubation of HER-2 Ab immobilized surface with untreated serum (**a**) sample 1, (**b**) sample 2, (**c**) sample 3, (**d**) sample 4, (**e**) sample 5, and (**f**) sample 6 of six breast cancer patients. Scale bar: 50 µm.

To determine the practicality and reliability of the purposed biosensor in clinical usage, real sample screening was carried out. According to the mentioned optimal assay protocol, the sensing platform was immobilized with 200 µg/mL HER-2 Ab and reacted with untreated serum samples from breast cancer cases. Clear birefringent signals with various interference colors are revealed via these real serum samples (Fig. 8). These findings confirmed the efficient clinical application of this assay.

**Figure 9:**
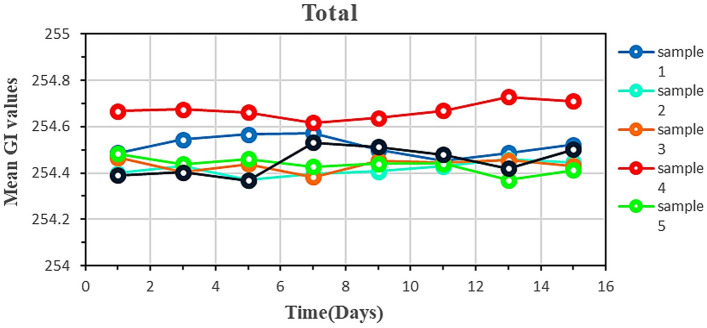
Mean GI values of breast samples in cancer patients as a function of time.

Besides studying the potential reliability of LC-based biosensing, it is important to examine the stability of the designed biosensors. To meet this goal, the alteration of the optical appearance of the proposed patient´s serum cells was assayed and their GIs were averaged over time. NO significant change in GI over 15 days (Fig. 9) highlighted adequate stability of the LC-based biosensors for practical applications.

## Conclusions

In this manuscript, we prosperously demonstrated a novel and reliable strategy for HER-2 biomarkers detection. The method is founded on a labeled free LC biosensor with a significant disruption of LC alignment after specific binding of HER-2 protein to the HER-2 Abs immobilized on DMOAP coated surface. This change was readily monitored from its optical appearance using POM, avoiding sophisticated laboratory equipment. For improving the binding affinity of the LC alignment agents, an easy UV irradiation approach was offered to provide oxygen functional groups that enhance binding toward HER-2 Abs. Moreover, the quantitative determination was carried out and a desirable linear correlation between mean GI values and HER-2 concentrations was noticed in the range of 10^–6^–10^2^ ng/mL. Having applied this sensing method, a highly sensitive biosensor down to 1 fg/mL for HER-2 detection limit was developed, which relies on the disturbance in the alignment and optical features of the LC molecules. The suggested LC biosensing approach not only permits a simple, rapid, and non-labeled detection for HER-2 with considerable sensitivity and specificity but also is anticipated for the HER-2 determination in real clinical samples with satisfying outcomes.

## Data Availability

All data generated or analysed during this study are included in this published article.
